# Detection of TRPV4 channel current-like activity in Fawn Hooded hypertensive (FHH) rat cerebral arterial muscle cells

**DOI:** 10.1371/journal.pone.0176796

**Published:** 2017-05-04

**Authors:** Debebe Gebremedhin, David X. Zhang, Dorothee Weihrauch, Nnamdi N. Uche, David R. Harder

**Affiliations:** 1 Department of Physiology, Medical College of Wisconsin, Milwaukee, Wisconsin, United States of America; 2 Cardiovascular Research Center, Medical College of Wisconsin, Milwaukee, Wisconsin, United States of America; 3 Department of Medicine and, Medical College of Wisconsin, Milwaukee, Wisconsin, United States of America; 4 Department of Anesthesiology Medical College of Wisconsin, Medical College of Wisconsin, Milwaukee, Wisconsin, United States of America; 5 Clement Zablocki VA Medical Center, Milwaukee, Wisconsin, United States of America; Indiana University School of Medicine, UNITED STATES

## Abstract

The transient receptor potential vallinoid type 4 (TRPV4) is a calcium entry channel known to modulate vascular function by mediating endothelium–dependent vasodilation. The present study investigated if isolated cerebral arterial myocytes of the Fawn Hooded hypertensive (FHH) rat, known to display exaggerated K_Ca_ channel current activity and impaired myogenic tone, express TRPV4 channels at the transcript and protein level and exhibit TRPV4-like single-channel cationic current activity. Reverse transcription polymerase chain reaction (RT-PCR), Western blot, and immunostaining analysis detected the expression of mRNA transcript and translated protein of TRPV4 channel in FHH rat cerebral arterial myocytes. Patch clamp recording of single-channel current activity identified the presence of a single-channel cationic current with unitary conductance of ~85 pS and ~96 pS at hyperpolarizing and depolarizing potentials, respectively, that was inhibited by the TRPV4 channel antagonist RN 1734 or HC 067074 and activated by the potent TRPV4 channel agonist GSK1016790A. Application of negative pressure via the interior of the patch pipette increased the NPo of the TRPV4-like single-channel cationic current recorded in cell-attached patches at a patch potential of 60 mV that was inhibited by prior application of the TRPV4 channel antagonist RN 1734 or HC 067047. Treatment with the TRPV4 channel agonist GSK1016790A caused concentration-dependent increase in the NPo of K_Ca_ single-channel current recorded in cell-attached patches of cerebral arterial myocytes at a patch potential of 40 mV, which was not influenced by pretreatment with the voltage-gated L-type Ca^2+^ channel blocker nifedipine or the T-type Ca^2+^ channel blocker Ni^2+^. These findings demonstrate that FHH rat cerebral arterial myocytes express mRNA transcript and translated protein for TRPV4 channel and display TRPV4-like single-channel cationic current activity that was stretch-sensitive and activation of which increased the open state probability of K_Ca_ single-channel current in these arterial myocytes.

## Introduction

Transient receptor potential vallinoid type 4 (TRPV4) channel, a member of the transient receptor potential (TRP) channel superfamily, is a non-selective cationic channel permeable to Na^+^, Ca^2+^, Mg^2+^ and is widely distributed in different cell types, including those of the brain [1, 2]. TRPV4 channel mainly operates as calcium entry pathway in cells in response to an array of physical perturbations, including pressure, flow, swelling, low pH, heat and changes in osmolality [3–8]. TRPV4 channel is also activated by substances such as anandamide, arachidonic acid and its CYP epoxygenase metabolites, the epoxyeicosatrienoic acids (EETs) that have been identified as endothelium-derived hyperpolarizing factors (EDHF) [9], and the phorbol ester derivative 4α-phorbol 12,13-didecanoate (4α-PDD) [3, 10, 11]. Changes in TRPV4 channel activity are involveed in diverse physiological functions including osmotic and volume regulation, thermo-sensing, mechanosensation in endothelium and urinary bladder, synaptic transmission, nociception, bone formation and remodeling [2, 12].

TRPV4 channel is extensively expressed in brain astrocytes and was identified to function as a key molecular sensor of hemodynamic stimuli and regulator of parenchymal arteriole tone [13]. Three axonal neuropathies including scapuloperoneal spinal muscle atrophy (SPSMA), congenital distal spinal muscle atrophy (CDSMA), and Charcot-Marie-Tooth disease type 2C (CMT2C) have been considered as allelic disorders caused by mutations in the gene encoding the TRPV4 channel [14]. Previous reports indicate that hypoxia/ischemia increases expression and activity of TRPV4 channel in brain astrocytes contributing to Ca^2+^ overload in the astroglial syncytium leading to cellular damage [15]. TRPV4 channels in endothelial and epithelial cells respond to mechanical force or flow-induced shear stress and mediate flow evoked vasodilation [10, 11, 16]. Despite the requirement for in-depth investigation of expression and kinetic properties, the presence of mRNA transcript for TRPV4 channel and an outwardly rectifying macroscopic cationic current that was activated by treatment with the TRPV4 channel agonist 4α-PDD has been previously reported in the Sprague Dawley rat strain cerebral arterial muscle cells [17]. In that study, TRPV4 was proposed to form a novel Ca^2+^ signaling complex with the ryanodine receptors and BK_Ca_ / K_Ca_ channel. Simulation of this complex with the epoxide 11,12-epoxyeicosatrienoic acid (11,12-EET) initiates smooth muscle hyperpolarization and vasodilation [17]. TRPV4 channel has also been reported to interact with PKC and AKAP150 (a kinase anchor protein 5) to form a dynamic signaling domains that control Ca^2+^ influx in arterial myocytes to oppose vasoconstriction in this rat strain [18].

The Fawn Hooded hypertensive (FHH) rat strain is a genetically created rat that displays disrupted cerebral and renal myogenic autoregulation of blood flow with exaggerated Ca^2+^-activated K^+^ (K_Ca_) channel current activity [19–21]. However, it is not known whether there is expression of a functional TRPV4 channel at transcript and protein levels. In addition, whether channels display TRPV4 channel biophysical and pharmacological properties, and whether they are sensitive to membrane stretch, and can increase the open state probability of K_Ca_ channel current following activation in the FHH rat cerebral arterial myocytes.

The present studies were undertaken: (1) to investigate expression of TRPV4 channel at the mRNA transcript and translated protein level, (2) to characterize TRPV4-like single-channel cationic current activity, [22] to examine the sensitivity of TRPV4 channel to membrane stretch (4) to determine its role in increased intraluminal pressure-induced cerebral arterial myogenic tone in FHH rats compared to that in Sprague Dawley rats and (5) to determine its functional influence on the open state probability of K_Ca_ single-channel current in the FHH rat isolated cerebral arterial myocytes using RT-PCR, Western blotting, immunofluorescence, the patch clamp channel current recording technique and pressure myograph.

The findings of the present studies revealed that TRPV4 channel is expressed at mRNA transcript and translated protein level in FHH rat cerebral arterial myocytes. This TRPV4 channel protein conducts a non-selective single-channel cationic current that is activated by membrane stretch and displayed pharmacological properties resembling that of heterologously expressed TRPV4 single-channel current. Treatment with the specific TRPV4 channel inhibitor HC067047 had no significant effect on increased intraluminal pressure-induced reductions in diameter of endothelium-denuded and cannulated FHH or Sprague Dawley rat cerebral arterial segments. Stimulation with the TRPV4 channel agonist GSK1016790A [23] resulted in increased opening probability of K_Ca_ single-channel current recorded in FHH rat cerebral arterial myocytes that was not influenced by inhibition of voltage–dependent Ca^2+^ channels.

## Material and methods

The animal protocols used in this study were approved by the Medical College of Wisconsin Institutional Animal Care and Use Committee (I.D. = AUA0000133). The studies were performed on male FHH rats (10–12 weeks of age) obtained from in bred colonies maintained at the Human Molecular Genetic Center (HMGC) of the Medical College of Wisconsin, which is approved by the American Association for the Accreditation of Laboratory Animal Care, and had free access to food and water throughout the study. The rats were anesthetized using 4% isoflurane and decapitated. Brains were quickly removed and placed on a dissecting dish containing ice-cold, low-calcium arterial muscle cell dissociation solution composed of (in mM): 134 NaCl, 5.2 KCl, 1.2 MgSO_4_.7 H_2_O, 1.18 KH_2_PO_4_, 0.05 CaCl_2_, 24 NaHCO_3_, 11 glucose, and 10 N-2-hydroxyethylpiperazine-N-2-ethanesulfonic acid (HEPES) (pH = 7.4). Cerebral arterial segments were carefully dissected out of the brain under a dissecting microscope and placed in a vial containing the dissociation solution.

### Enzymatic dispersion of FHH rat cerebral arterial segments to myocytes

Freshly isolated FHH rat cerebral arterial segments were placed in a vial containing 2 ml solution of bovine serum albumin (0.5 mg/ml) in the low-calcium arterial muscle cell dissociation solution for 10 min at room temperature. These arterial segments were then transferred to another vial containing 1 ml solution of dithiothreitol (Sigma, St Louis, MO, USA) (0.5 mg/ml) and papain (Worthington, Freehold, NJ, USA) (60 Units/ml) in low-calcium dissociation solution, and placed in a water-jacketed beaker (at 37°C) and incubated for 20 min. After incubation, the supernatant layer was removed and replaced with 0.5 ml of dissociation solution containing collagenase type II (0.5 mg/ml; Worthington, Freehold, NJ, USA) and trypsin inhibitor (0.1 mg/ml, Sigma, St Louis, MO, USA), and incubated at 37°C (pH = 7.4). Supernatant fractions were collected at 5 min intervals and each fraction was diluted to 2 ml with fresh low-calcium dissociation solution. The procedure was repeated by incubating the remaining cerebral arterial tissue with fresh enzyme containing low-calcium dissociation solution. Complete dispersion of the cerebral arterial segments to single arterial muscle myocytes was typically attained within 30–40 min of incubation. The dissociated cerebral arterial myocytes were used for immunofluorescence, RT-PCR, Western blot analysis, and for patch clamp recordings of TRPV4 or K_Ca_ single-channel currents under different experimental conditions in this study.

### RNA extraction and RT-PCR

RT-PCR was performed on freshly dissociated FHH rat isolated cerebral arterial myocytes by modification of previously described method [24]. Briefly, total RNA was extracted from freshly dissociated cerebral arterial myocytes using the RNeasy High Capacity cDNA Reverse Transcription Kit (Applied Biosystems) with random primers to generate cDNA with 1:2 dilutions to serve as template for quantitative real time PCR (qPCR). The absence of contamination by endothelial cells of a population of dissociated cerebral arterial myocytes used for RT-PCR, Western immunoblot analysis or patch clamp single-channel current recording were used after identification of the cells by a negative immunostaining with the endothelial cell marker anti-CD31 or PECAM-1 antibody and by intense positive immunostaining with anti-smooth muscle α-actin antibody. The gene-specific primer for qPCR had the following sequence: forward, 5’-CCA AGG ATG AGG GTG GCT-3’; and reverse, 5’-GTC GGA TGA TGT GCT GAA AG-3’ (TRPV4_Rattus accession #: NM_023970). The TaqMan Real-time PCR was performed using Bullseye TaqProbe qPCR MasterMix-Low ROX (MIDSci) with a final concentration of 0.4 μM of the primer. The cycling conditions included an initial incubation step at 95°C for 3 min. Amplification was then performed using 39 cycles of denaturation at 95°C for 30 s and annealing and extension at 60°C for 1 min. PCR products were electrophoresed in a 0.8% agarose gel, stained with ethidium bromide, and visualized under UV light.

### Western blot analysis

The expression of TRPV4 channel protein in freshly dissociated FHH rat cerebral arterial myocytes was examined by Western blot analysis. Protein samples were prepared from freshly dissociated FHH rat cerebral arterial myocyte, and also from whole brains of FHH rat and the control Sprague Dawley rat that display normal myogenic cerebral autoregulation, by homogenization in lysis solution (Camiolo buffer, 75 mM potassium acetate, 300 mM NaCl, 10 mM EDTA, 100 mM l-arginine basic salt, and 0.25% Triton-X 100, protease inhibitor mix). Protein concentrations were determined using the Bradford assay [25]. Proteins were separated on 10% sodium dodecyl sulfate polyacrylamide gel electrophoresis and transferred to a membrane (Immobilon-P polyvinylidene difluoride, Millipore) at 25°C for 2 h at 15 V. Following 2 h blocking (with 5% skim milk in Tris-buffered saline with Tween-20 (TBST), the membrane was then incubated with polyclonal TRPV4 primary antibodies (ACC-034, 1:500, Alomone Laboratories) overnight at 4°C, and then with horseradish peroxidase (HRP)-conjugated goat anti-rabbit polyclonal secondary antibody (DakoCytomation; 1:1,500) for 1 h at room temperature. An ECL Plus Detection kit (Amersham Biosciences) was used to visualize bands.

### Effect of TRPV4 inhibition on pressure-induced myogenic reactivity of cerebral arterial segments

Male FHH rats or Sprague-Dawley rats (10–12 weeks of age) obtained from Charles River were anesthetized with 4% isoflurane and decapitated after the disappearance of corneal reflexes. Brains were quickly removed and placed on a dissecting dish containing ice-cold physiological salt solution (PSS) of the following composition (mM): NaCl (141), KCl (4.7), CaCl_2_ (2.5), MgCl_2_ (0.72), NaH_2_PO_4_ (1.7), NaHCO_3_ (25), glucose (11), EDTA (0.015) and HEPES (10). Cerebral arterial segments were carefully dissected from the brain under a dissecting microscope and placed in PSS in a dish. Both ends of the isolated cerebral arterial segments were cannulated on glass pipettes tied in place with 8–0 sutures in a pressure myograph containing PSS and the pH maintained between 7.4–7.5 by bubbling with 95% O_2_ and 5% CO_2_ and checked at the beginning of each experiment using a blood gas analyzer (ABL80 FLEX, Radiometer Medical ApS, Denmark). The internal diameter of the cannulated cerebral arterial segments was measured using a Living Systems Video Dimension Analyzer, chamber and pressure servo control (Systems Instrumentation, Burlington, VT, USA). The cannulated cerebral arterial segments were pressurized at 60 mm Hg and equilibrated for 60 min while being superfused with PSS bubbled with a mixture of 95% O_2_ and 5% CO_2_. The endothelium lining the cerebral arterial segments was removed by gentel passage of a bolus of air through the lumen for 1 to 2 minutes. Removal of the endothelium was validated by the absence of dilation of serotonin (2 μM) constricted cannulated cerebral arterial segments in response to addition of 1 μM acetylcholine to the bath (data not shown). The arterial segments were repeatedly washed with fresh PSS and the intraluminal pressure adjusted to 20 mm Hg. The cannulated cerebral arterial segments were then pressurized from 20 mm Hg to 100 mm Hg in in steps of 20 mm Hg and the inner diameter was measured every 1 and 5 minutes at each pressure level. The effects of treatment with the TRPV4 channel inhibitor HC067047 (1 μM) applied to the bath was examined after a minimum of 5 min prior to repeat of measurement of pressure-induced changes in inner arterial diameter.

### Immunofluorescence microscopy in single isolated cerebral arterial myocytes

To adhere freshly isolated FHH rat cerebral arterial myocytes to slides for immunofluorescence studies, 250 microliters of cells suspension was pipetted into a single cytofunnel (Shandon, Pittsburgh, PA) and spun onto coated slides (Fisher Scientific, Staining control slides, Cat. No. 22-042-910) at a speed of 1000 rpm for 5 min at room temperature in a cytospin (Shandon Cytospin 3). After the cytospin, the cells were fixed in ice-cold acetone for 5 min. Acetone was removed and the adhered cells were then labeled with primary antibody mouse anti-rat monoclonal CD31 (#550300, BD Biosciences), rabbit polyclonal TRPV4 (#ACC-034, Alomone labs) or mouse monoclonal anti-actin, α-Smooth Muscle-Cy3 conjugated (#C6198, Sigma Aldrich) for 30 min at 37°C. After the 30 min incubation period, the slides were gently washed once in phosphate buffered saline (PBS) for three min. The secondary antibody Donkey anti-Rabbit Alexa Fluor 488 (ab150073, Abcam) was used at a 1:1000 dilution in PBS and applied to the slides for 30 min at 37°C. A final three minute wash step was performed with PBS containing DAPI at a 1:1000 dilution. The slides were sealed with an aqueous mounting media (TBS Shur Mount, VWR) and stored in the refrigerator overnight. Confocal microscopy employed was a Nikon Eclipse TE 200-U (Nikon, Japan) equipped with EZ C1 laser scanning software. Images were captured using a 30 μm emission pinhole with a 20X or 60X objective using excitation of 488 nm for TRPV4 and CD31, 418 nm for nuclear labeling with DAPI and 533 nm for α-Smooth Muscle actin.

### TRPV4 immunofluorescence in cerebral arterial sections

FHH rat cerebral arterial segments were fixed in 4% paraformaldehyde for 24 h and then paraffin embedded. The embedded arterial segments were sectioned at 4 μM thickness and dried on glass slides at 45°C overnight. The sectioned samples were antigen retrieved with DAKO target retrieval solution (pH = 6, DakoCytomation, s1699) for 40 min at 99°C followed by cool down at room temperature for 20 min. The slides were then rinsed with TBST and protein blocked for 30 min with the reagent removed by blow off. Polyclonal anti-TRPV4 antibody (Alomone ACC-034) was applied to the slides at a dilution of 1:200 and incubated overnight at 4°C. The slides were then rinsed twice with TBST for 5 min and treated with a secondary antibody, Donkey anti-Rabbit Alexa 488 (ab150073, Abcam), at a dilution of 1:750 for 45 min at room temperature. The slides were then rinsed twice with TBST for 5 min and counterstained with DAPI at a dilution of 1:1000 for 10 min followed by a rinse with distilled water and covered with ProLong Gold antifade mounting medium (Life Technologies) for 30 min and sealed with nail polish. Images were captured with AIM 4.2 software controlling Zeiss LSM510 microscope (Jena, Germany) and 40 x objectives.

### Patch clamp recording of single-channel currents

Single-channel currents were recorded at room temperature from cell-attached membrane patches of freshly isolated FHH rat cerebral arterial myocytes using the patch-clamp technique as described previously [19, 26, 27]. Briefly, recording pipettes were fabricated from borosilicate glass, pulled on a 2-stage micropipette puller (P-97), and heat-polished under a microscope (MF-83 heat polisher; Narishige, Tokyo, Japan). The recording pipettes were mounted on a three-way hydraulic micromanipulator (Narishige) for placement of the tips on the cell membrane. High-resistance seals (>1 GΩ) were established by applying a slight suction between fire-polished pipette tips (3–10 MΩ) and arterial myocyte membranes. The offset potentials between pipette and bath solution were corrected with an offset circuit before each experiment. Single-channel currents were recorded using an Axopatch 200B amplifier (Molecular Devices, CA). The amplifier output was low-pass filtered at 1 kHz. Current signals were digitized at a sampling rate of 10 kHz (Digidata 1440A, Molecular Devices, CA). Single-channel currents were analyzed using a pClamp software package (pClamp version 10.4; Molecular Devices, CA) to determine opening frequency, mean current amplitudes, and open state probability. The mean open state probability (NPo) was expressed as NPo = I/i, where I is the time averaged current, N is the number of channels, i is the amplitude of the unitary current, and Po is the probability of a channel being open.

### TRPV4-like single-channel current recording

For recording of TRPV4-like single-channel cationic currents from cell-attached patches of freshly isolated FHH rat cerebral arterial myocytes, the pipette and bath recording solutions contained symmetrical 145 mM KCl (assuming 145 mM KCl inside cells) to bring the equilibrium for K^+^ and the cell resting membrane potential to 0 mV to minimize contribution of possible voltage artifacts. Thus, the recording pipette or electrode solution contained (in mM) 145 KCl, 1 MgCl_2_, and 10 HEPES adjusted to pH 7.4 with CsOH, and the bath solution was composed of (in mM) 145 KCl, 0.8 CaCl_2_, 1 MgCl_2_, 5 glucose, 5 EGTA, and 5 HEPES adjusted to 7.3 with CsOH. The final Cs^+^ concentration in the bath solution was 4 mM [28]. Single-channel currents were recorded from cell-attached patches of FHH rat cerebral arterial myocytes at different patch potentials (difference between plasma membrane potential of 0 mV under symmetrical 145 mM KCl solution recording condition and the externally applied pipette potential in mV) starting from -100 mV to 100 mV in steps of 20 mV. The patch potential (60 mV) at which moderate single-channel cationic current opening was observed was chosen to study the effects of specific pharmacological antagonist of the TRPV4 channel HC 067047 or RN 1734 [29–31] (Tocris Biosciences) and the TRPV4 channel agonist GSK1016790A [23] (Sigma-Aldrich, MO) that have been previously shown to selectively inhibit or activate TRPV4 channel current, respectively, in expression systems [23, 29, 30]. In additional studies, the effect of localized and stepped increase in membrane stretch achieved by applying suction (negative pressure) with a calibrated glass syringe to the back end of the patch electrode through the suction port of the electrode holder by modification of a method previously described [32, 33]. The level of pressure was monitored continuously with a connected calibrated water manometer and pressure transducer. The effect of membrane stretch on the openings of the single–channel currents during recording was examined before and after treatment of the cell-attached patches with the TRPV4 channel inhibitor HC 067047 (300 nM) or RN 1734 (5 μM) every 1 min for a duration of 5 min. All Patch clamp recordings of single-channel membrane currents were performed at room temperature. To determine the unitary conductance of the TRPV4-like single-channel cationic current in the FHH rat cerebral arterial myocytes, a linear regression plot of different patch holding potentials ranging from -100 mV to +100 mV in steps of 20 mV versus averaged unitary cationic current amplitudes (pA) recorded at each potential was constructed and the slope conductance calculated.

### Single-channel Ca^2+^-activated K^+^ (K_Ca_) current recording

For patch clamp recording of K_Ca_ single-channel currents from cell-attached patches of FHH rat cerebral arterial myocytes the **r**ecording pipette solution contained (in mM): KCl 145, CaCl_2_ 1.8, MgCl_2_ 1.1, HEPES 5, ethylene-glycol-bis (β-aminoethyl ether)-N, N, N’, N’-tetra acetic acid (EGTA) 5, with the final pH adjusted to 7.2 with KOH. The bath solution was composed of (in mM): KCl 145, CaCl_2_ 1.8, MgCl_2_ 1.1, HEPES 5, EGTA 5, with pH adjusted to 7.2 with KOH. This resulted in a calculated final bath [Ca^2+^] of 10^-7^M as previously described [26, 27]. The single-channel K_Ca_ current recorded from cell-attached patches of FHH rat cerebral arterial myocytes had a unitary slope conductance of 258 ± 5 pS when determined using symmetrical KCl (145 mM) recording solution over a voltage range of -40 mV and +80 mv in 20 mV increments and was inhibited by the K_Ca_ channel blocker paxilline (1 μM). To determine if specific stimulation of TRPV4 channel expressed in cerebral arterial myocytes influences the open state probability of native K_Ca_ single-channel current, single-channel K_Ca_ currents were recorded at room temperature from cell-attached patches of freshly dissociated FHH rat cerebral arterial myocytes at a patch potential of +40 mV before and after treatment with the TRPV4 channel agonist GSK1016790A (10 and 100 nM). These studies were performed in the absence and presence of the K_Ca_ channel inhibitor 1 μM paxilline [22], the L-type Ca^2+^ channel inhibitor nifidepine (1 μM) or the T-type Ca^2+^ channel inhibitor nickel (50 μM) [34, 35] as previously described [26, 27].

### Drugs

All chemicals used were analytical grade. GSK1016790A (N-((1S)-1-{[4-((2S)-2-{[(2,4-Dichlorophenyl)sulfonyl]amino}-3-hydroxypropanoyl)-1-piperazinyl] carbonyl}-3-methylbutyl)-1-benzothiophene-2-carboxamide), nifedipine, serotonin, acetylcholine, and nickel were obtained Sigma-Aldrich (St. Louis, MO). Paxilline, RN 1734 (2,4-Dichloro-*N*-isopropyl-*N*-(2-isopropylaminoethyl) benzenesulfonamide) and HC 067047 (2-Methyl-1-[3-(4-morpholinyl)propyl]-5-phenyl-*N*-[3-(trifluoromethyl)phenyl]-1*H*-pyrrole-3-carboxamide) were obtained from Tocris Biosciences (Bristol, UK). Stock solutions of GSK1016790A, HC 067047, RN 1734, paxilline were prepared in dimethylslfoxide (DMSO, Sigma-Aldrich, St. Louis, MO). All concentrations represent final molar concentrations in the bath. The final concentration of the vehicle DMSO in the bath was <0.1%; and had no effect on activities of the single-channel currents recorded from the cell-attached patches of the FHH rat cerebral arterial myocytes.

### Statistics

Data are presented as mean values ± SEM. Differences in mean values between groups were assessed using Student's *t* test or one-way analysis of variance (ANOVA) for multiple comparisons followed by a Duncan’s new multiple range tests. A *P* value < 0.05 was considered statistically significant.

## Results

### Detection of expression of mRNA transcript and protein for TRPV4 channel in FHH rat cerebral arterial myocytes

**Fig 1:**
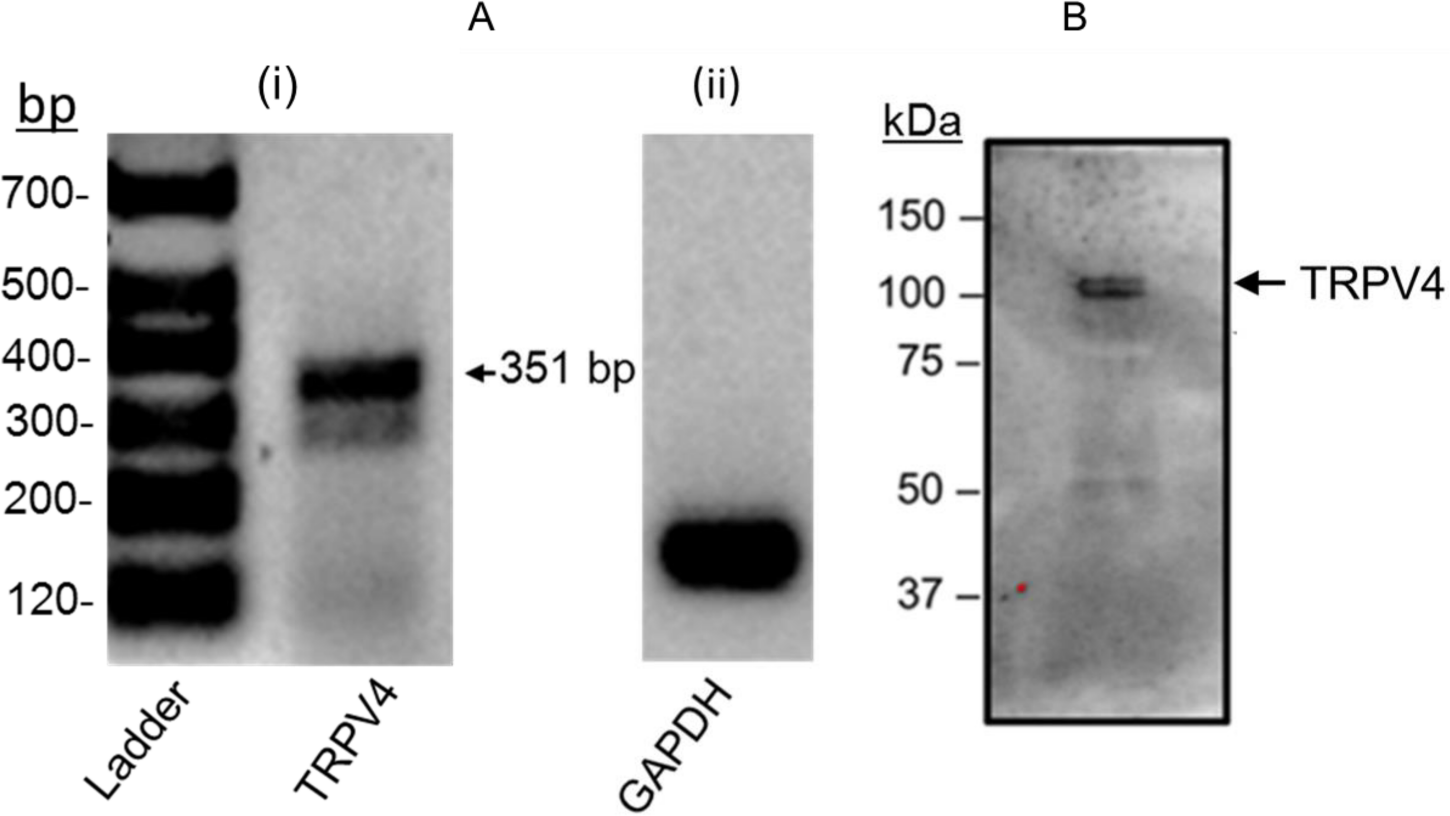
TRPV4 mRNA transcript and protein expression in FHH rat cerebral arterial myocytes. (A) (i) Use of TRPV4 channel gene specific primer amplified a PCR product of 351-bp as determined by semiquantitative RT-PCR. Shown in (ii) is the internal control GAPDH. (B) Western blot analysis using homogenates of freshly isolated FHH rat cerebral arterial myocytes lysates and the polyclonal anti-TRPV4 antibody (Alomone ACC-034 at 1:200) detected expression of the right molecular size for TRPV4 channel protein (n = 2–3 separate trials for each group).

The present studies of RT PCR analysis using gene specific primer amplified a PCR band of expected size (351 bp) for TRPV4 channel and the internal standard GAPDH in freshly dissociated FHH rat cerebral arterial myocytes (Fig 1A (i), (ii)). Furthermore, as depicted in Fig 1B, results of Western blot analysis of homogenates of freshly dissociated FHH rat cerebral arterial myocyte lysate using polyclonal antibody against TRPV4 channel identified presence of a protein band of the expected molecular size for TRPV4 channel protein.

**Fig 2:**
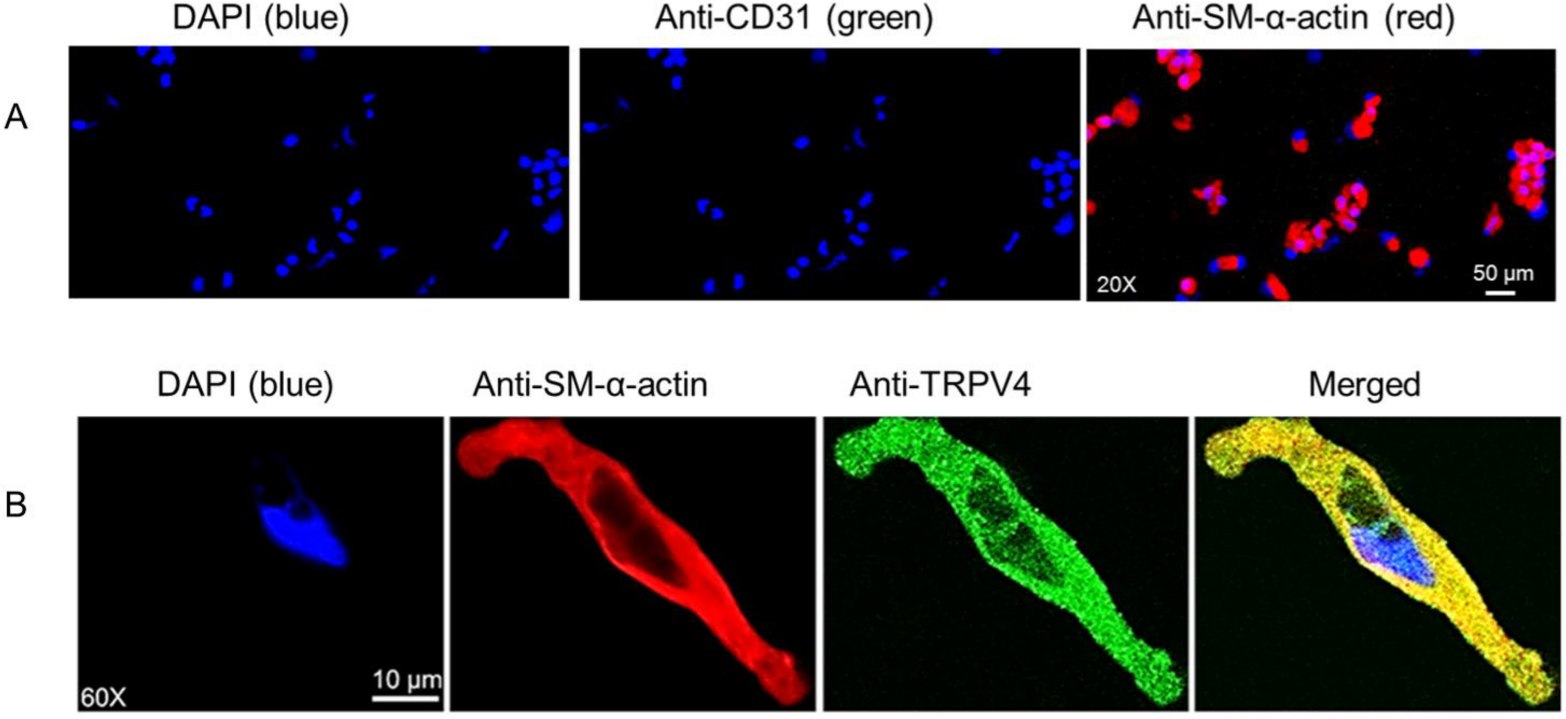
Differentiation by immunofluorescent staining of a population of freshly isolated FHH rat cerebral arterial myocytes and immunolocalization of TRPV4 channel protein expression. (A) Characterization and differentiation by immunofluorescent staining of a population of freshly isolated cerebral arterial myocytes using the nuclei stain DAPI, the endothelial cell marker anti-CD31 (PECAM-1) antibody and anti-smooth muscle α-actin (Anti-SM-α-actin) antibody visualized with donkey anti-rabbit Alexa Fluor 488-conjugated secondary antibody. These findings revealed that population of freshly isolated cerebral arterial myocytes obtained using the method described in this study are smooth muscle α-actin positive cells that are free of endothelial cell contamination. (B) Depiction of single freshly isolated rat cerebral arterial myocyte stained with DAPI alone (nuclei stained blue) or co-stained with either anti-SM-α-actin antibody (red) or with anti-TRPV4 antibody [36] and visualized with donkey anti-rabbit Alexa Fluor 488-conjugated secondary antibody. The image of the arterial myocyte stained with anti-TRPV4 antibody demonstrates expression of TRPV4 channel protein in the cerebral arterial myocyte positively stained with anti-SM-α-actin antibody.

Immunofluorescent staining studies of freshly dissociated FHH rat cerebral arterial myocytes (Fig 2A) revealed that a population of freshly isolated cerebral arterial myocytes obtained using the method described in the present study are smooth muscle α-actin positive cells (red) that are stained blue with DAPI (nuclei stain), and are free of endothelial cell contamination as confirmed by negative staining of the cells with the endothelial cell marker anti-CD31-antibody (middle panel). Fig 2B depicts single freshly isolated rat cerebral arterial myocyte stained with DAPI alone (nuclei stained blue) or stained with either anti-SM-α-actin antibody (red) or with anti-TRPV4 antibody [36] and visualized with donkey anti-rabbit Alexa Fluor 488-conjugated secondary antibody. The cerebral arterial myocyte stained with anti-SM-α-actin antibody was also intensely stained with anti-TRPV4 antibody identifying the expression of TRPV4 channel protein in the arterial myocytes.

**Fig 3:**
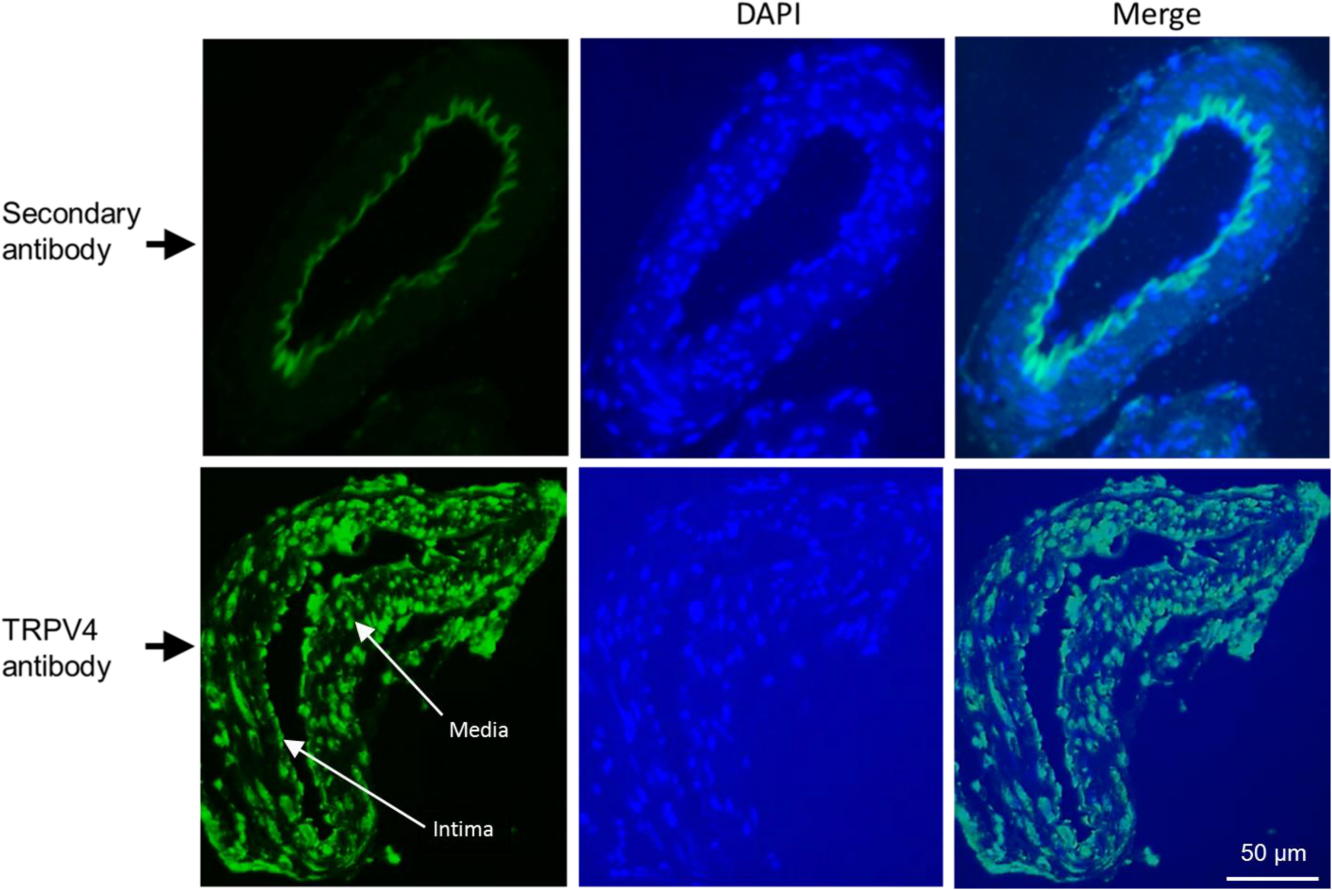
TRPV4 channel protein expression in cerebral arterial sections. Immunohistochemically detection of expression of TRPV4 channel protein using 4 μm sections of FHH rat cerebral arterial segment by treatment with either a secondary antibody alone (secondary antibody Donkey anti-Rabbit Alexa Fluor 488, 1:750, Thermo Fisher Scientific, upper panel) or with a primary polyclonal anti-TRPV4 antibody (Alomone ACC-034, 1:200, lower panel) revealing expression of TRPV4 protein in FHH rat cerebral arterial muscle cells as shown by punctate staining pattern both at the intima and media layers of the arterial wall. Blue: indicates nuclear staining with DAPI of the fixed arterial sections.

The merged images of FHH rat cerebral arterial myocyte stained with anti-SM-α-actin antibody (red) and anti-TRPV4 antibody [36] appeared in mixed colors of yellow and green demonstrating expression and co-localization of smooth muscle α-actin and TRPV4 channel protein exhibiting a punctate like pattern both in the cytosol and the cell membrane. As shown in Fig 3, additional immunofluorescence assay using 4 μm sections of FHH rat cerebral arterial segment by treatment with either a secondary antibody alone (secondary antibody Donkey anti-Rabbit Alexa Fluor 488, 1:750, Thermo Fisher Scientific, upper panel) or together with a primary polyclonal anti-TRPV4 antibody (Alomone ACC-034, 1:200, lower panel) detected expression of TRPV4 channel protein in FHH rat cerebral arterial muscle cells as shown by punctate staining pattern indicated with white line arrows both at the intima and media layers of the arterial wall sections.

### Identification of TRPV4-like single-channel cationic currents in the FHH rat cerebral arterial myocytes

**Fig 4:**
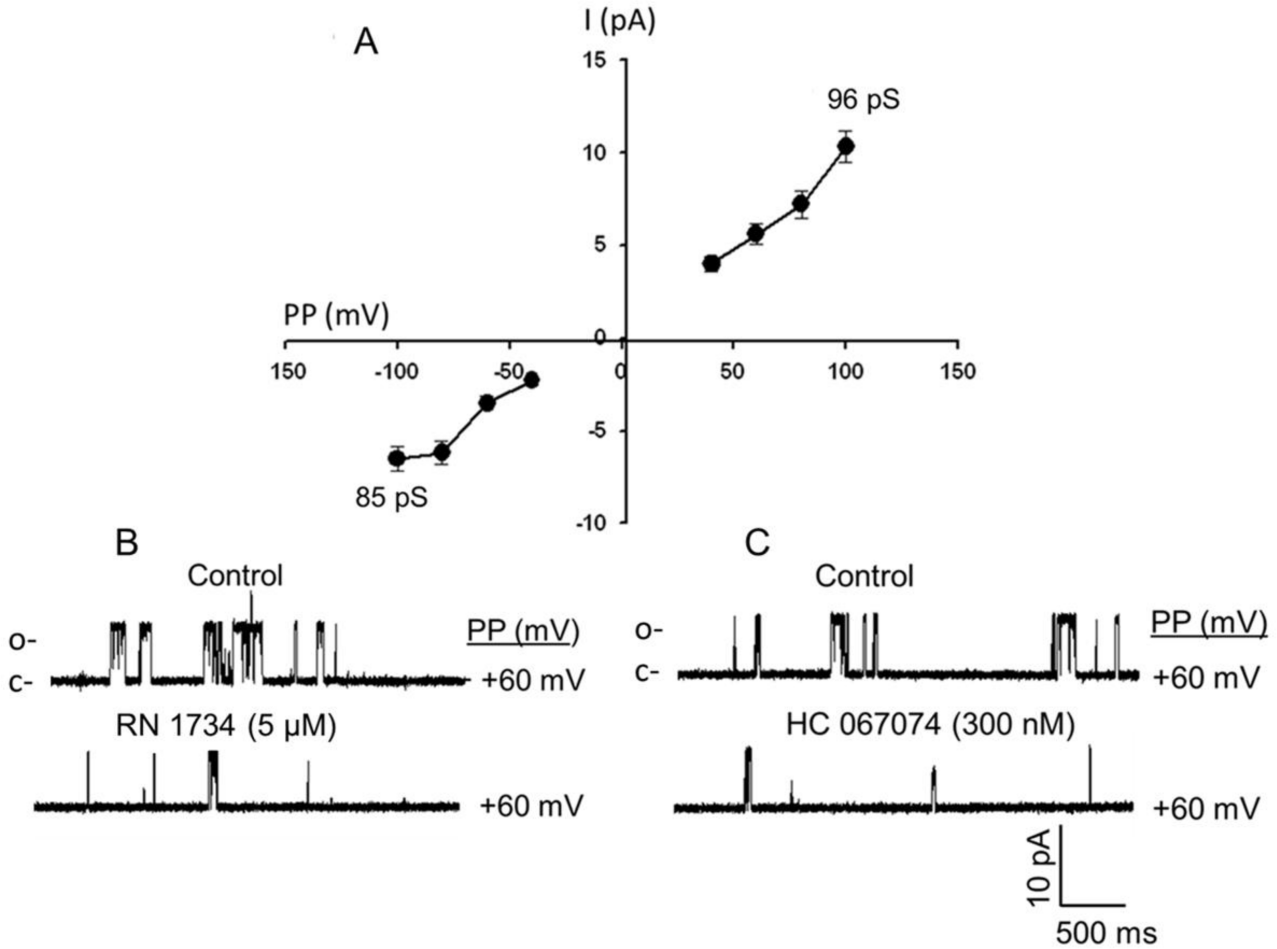
TRPV4-like single-channel cationic current conductance. Single-channel cationic currents recorded from FHH rat cerebral arterial myocytes exhibited two slope conductances: 85 ± 2 pS at more hyperpolarizing potentials and 96 ± 3 pS at more depolarizing potentials. (A) The single-channel cationic currents recorded at patch holding potential (PP) of 60 mV were sensitive to inhibition by the TRPV4 channel antagonist RN 1734 (5 μM) (B) or by HC 067047 (300 nM) (C) (n = 5–6 cells).

The patch clamp single-channel current analysis of the present studies characterized activities of TRPV4-like single-channel cationic current in cell-attached patches of FHH rat cerebral arterial myocytes. The recorded single-channel currents displayed an inwardly rectifying current with unitary conductance of 85 ± 2 pS at hyperpolarizing patch potentials and an outwardly rectifying current with unitary conductance of 96 ± 3 pS (n = 5–6 cells) at depolarizing patch potentials (Fig 4A). Channel activity was detected in 60% of the cell-attached patches (6/10). The single-channel cationic currents recorded from cell-attached patches at a patch potential of 60 mV were significantly inhibited by treatment with the TRPV4 channel inhibitor RN 1734 (5 μM) (Fig 4B) or HC 067047 (300 nM) (Fig 4C).

**Fig 5:**
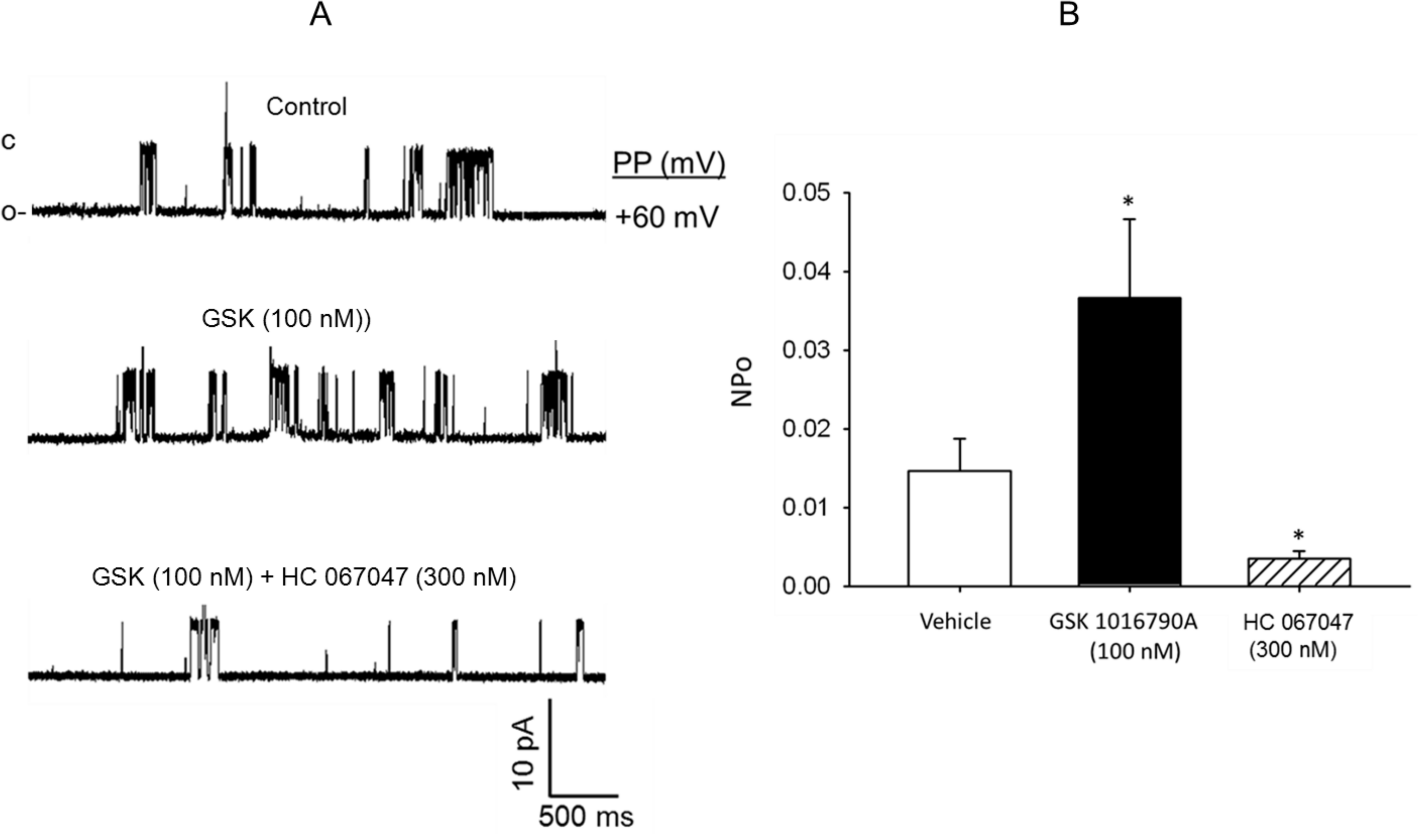
Characterization of the TRPV4-like single-channel cationic currents. The TRPV4-like cationic single-channel current recorded in cell-attached patches of FHH rat cerebral arterial myocytes was activated by the specific TRPV4 channel agonist GSK1016790A (100 nM) that was reduced or suppressed by the TRPV4 channel inhibitor HC 067047 (300 nM) (A and B). * denotes statistically significant difference from control at P < 0.05, n = 5–6 cells).

In a separate group of studies, stimulation of the cell-attached patches with the specific TRPV4 channel agonist GSK1016790A at a concentration of 100 nM by addition to the bath induced an increase in the NPo of the single-channel cationic currents from the control value of 0.014 ± 0.0041 to 0.036 ± 0.012 (P < 0.05, n = 5–6 cells) that was significantly reduced to 0.00345 ± 0.00098 (P < 0 < 0.05, n = 5–6 cells for each group) following addition of the TRPV4 channel inhibitor HC 067047 (300 nM) to the bath (Fig 5A and 5B). These findings demonstrate that the FHH rat cerebral arterial myocytes express a single-channel cationic current that display unitary slope conductance and pharmacological properties similar to that reported for expressed TRPV4 single-channel cationic currents [30, 31, 37, 38].

### Effect of membrane stretch on TRPV4-like single-channel cationic current activity in FHH rat cerebral arterial myocytes

**Fig 6:**
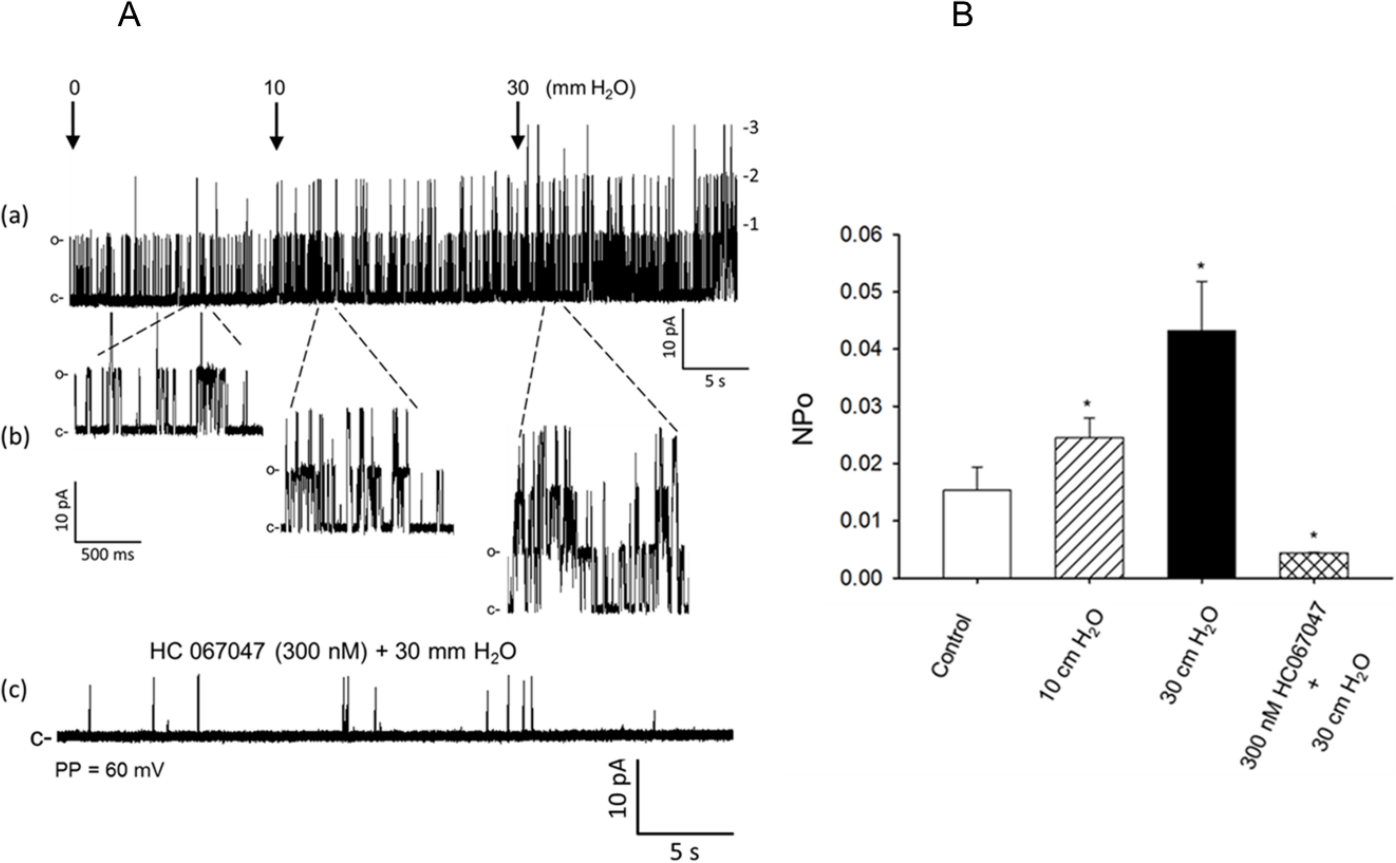
Sensitivity of TRPV4-like single-channel cationic current to membrane stretch. (A) Representative single TRPV4-like single-channel cationic currents recorded at a holding potential of 60 mV from cell attached patches of cerebral arterial myocytes shown in slower (a) and faster time spans (b) under control condition and following membrane stretch by application of negative pressure of 10 mm H_2_O or 30 mm H_2_O and (c) attenuation of the 30 mm H_2_O negative pressure induced activation of the single-channel cationic current by pretreatment with the TRPV4 channel inhibitor HC 067047. (B) Summary of application of a negative pressure of 10 cm H_2_O or 30 cm H_2_O induced marked increase in the open state probability of (NPo) of the TRPV4-like single-channel cationic current and the attenuation of the negative pressure of 30 mm H20 induced increase in NPo following treatment with the TRPV4 channel inhibitor HC 067047 (300 nM) (*, ^†^, P < 0.05, n = 5–6 cells for each group).

Application of suction induced negative pressure of 10 cm H_2_O or 30 cm H_2_O to the recording patch pipette interior caused an increase in the NPo of the TRPV4 single-channel cationic current recorded at patch potential of 60 mV from the control value of 0.0153 ± 0.0049 to 0.0245 ± 0.00341 at 10 cm H_2_0 (by ~37%), and to 0.0432 ± 0.006 at 30 cm H_2_0 (by ~65%) (*P < 0.05, Fig 6A and 6B, n = 5–6 cells). In these studies, however, increasing the magnitude of suction induced negative pressure through the interior of the recording pipette at levels greater than 30 cm H_2_O resulted in a marked decrease in seal resistance from ~3–6 gigaohm (GΩ) to less than ~300 megaohm (mΩ) rendering optimal recordings of single-channel cationic currents virtually impossible. For these reasons, studies of the effects of negative pressure induced local membrane stretch on TRPV4-like single-channel cationic current opening frequency and NPo was examined only at negative pressures of 10 cm H_2_O and 30 cm H_2_O. In these studies, exposure of the cell-attached patches to the TRPV4 channel inhibitor HC 067047 (300 nM) significantly reduced the 30 cm H_2_0 negative pressure induced increased opening frequency and NPo from 0.0432 ± 0.006 to 0.0044 ± 0.00012 (*P < 0.05, n = 5–6 cells) of the TRPV4-like single-channel currents recorded at a patch potential of 60 mV (Fig 6B). The amplitude of the single-channel cationic current studied was not altered by application of any of the negative pressure levels studied (data not shown).

### Effects of the specific TRPV4 channel agonist GSK1016790A on single-channel K_Ca_ currents in FHH rat cerebral arterial myocytes

**Fig 7:**
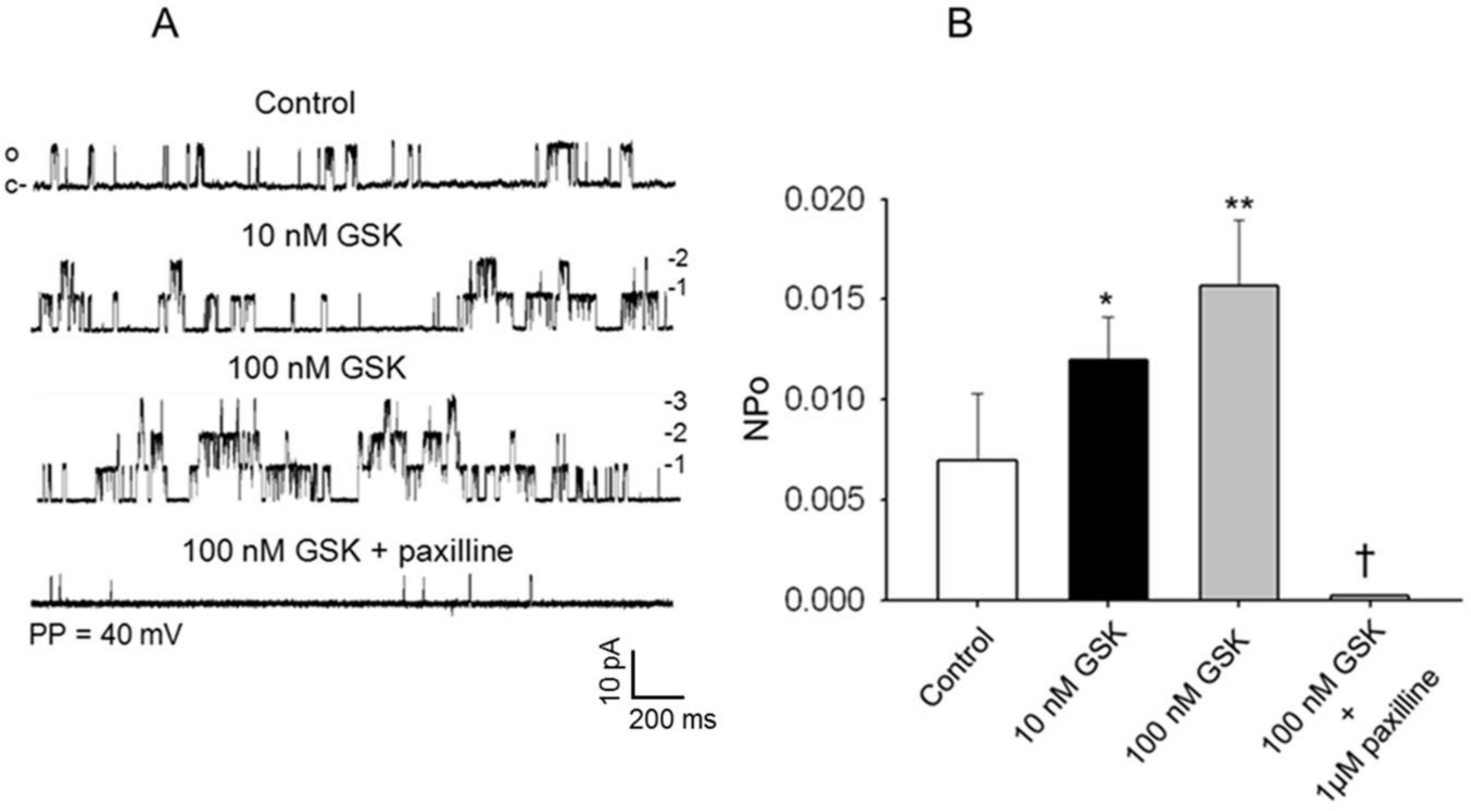
Effect of the TRPV4 channel agonist GSK1016790A on activities of a K_Ca_ single-channel current in FHH rat cerebral arterial myocytes. (A) Examples of single-channel current traces recorded at a patch holding potential of 40 mV using symmetrical (145 mM KCl) recording solution under control condition and following treatment with 10 nM or 100 nM GSK1016790A. Application of GSK1016790A induced concentration-dependent increase in the opening frequency of the single-channel K_Ca_ currents that was attenuated by treatment with the K_Ca_ channel blocker paxilline. (B) Summary of the effects of treatment with 10 nM or 100 nM GSK1016790A on the open state probability (NPo) of the K_Ca_ single-channel currents recorded at a patch holding potential of 40 mV. Treatment with 10 nM or 100 nM GSK1016790A significantly increased the NPo of the K_Ca_ single-channel currents compared to the control NPo value that was attenuated by treatment with the K_Ca_ channel inhibitor paxilline. (*,† denote P < 0.05, n = 5–6 cells for each group; c and o represent closed and open states of the channel, and 1,2,3 indicate stocked channel opening levels).

The intent of these studies was to examine the influence of TRPV4 channel activation on the opening probability of a 258 pS native K_Ca_ single-channel current in cerebral arterial myocytes. Thus, the effects of stimulation of native TRPV4 channel current with its specific agonist GSK106790A on the opening frequency and NPo of K_Ca_ single-channel current recorded from cell-attached patches of cerebral arterial myocytes at a patch potential of 40 mV using symmetrical 145 mM KCl solution was examined. In these studies, application of the TRPV4 channel agonist GSK1016790A at a concentration of 10 nM or 100 nM to the bath induced a significant concentration-dependent increase in opening frequency as well as the NPo of the single-channel K_Ca_ current from a control value of 0.00697 ± 0.003 to 0.01614 ± 0.0032 (* P < 0.05, Fig 7, n = 6 cells) during recording from cell-attached patches at a patch potential of 40 mV that was reduced to 0.00025 ± 0.0005 following addition to the bath the selective K_Ca_ channel current inhibitor 1 μM paxilline [22], (Fig 7A and 7B, *, ** P < 0.05, and † P < 0.001, n = 5–6 cells). These results demonstrate the capacity of specific stimulation of TRPV4 channel to evoke an increase in activities of a single-channel K_Ca_ current suggestive of presence of possible functional interaction between TRPV4 channel and K_Ca_ channel activities in the FHH rat cerebral arterial myocyte.

### Influence of voltage-dependent Ca^2+^ channel modulation on the TRPV4 channel agonist GSK1016790A induced activation of K_Ca_ single-channel current in FHH rat cerebral arterial myocytes

**Fig 8:**
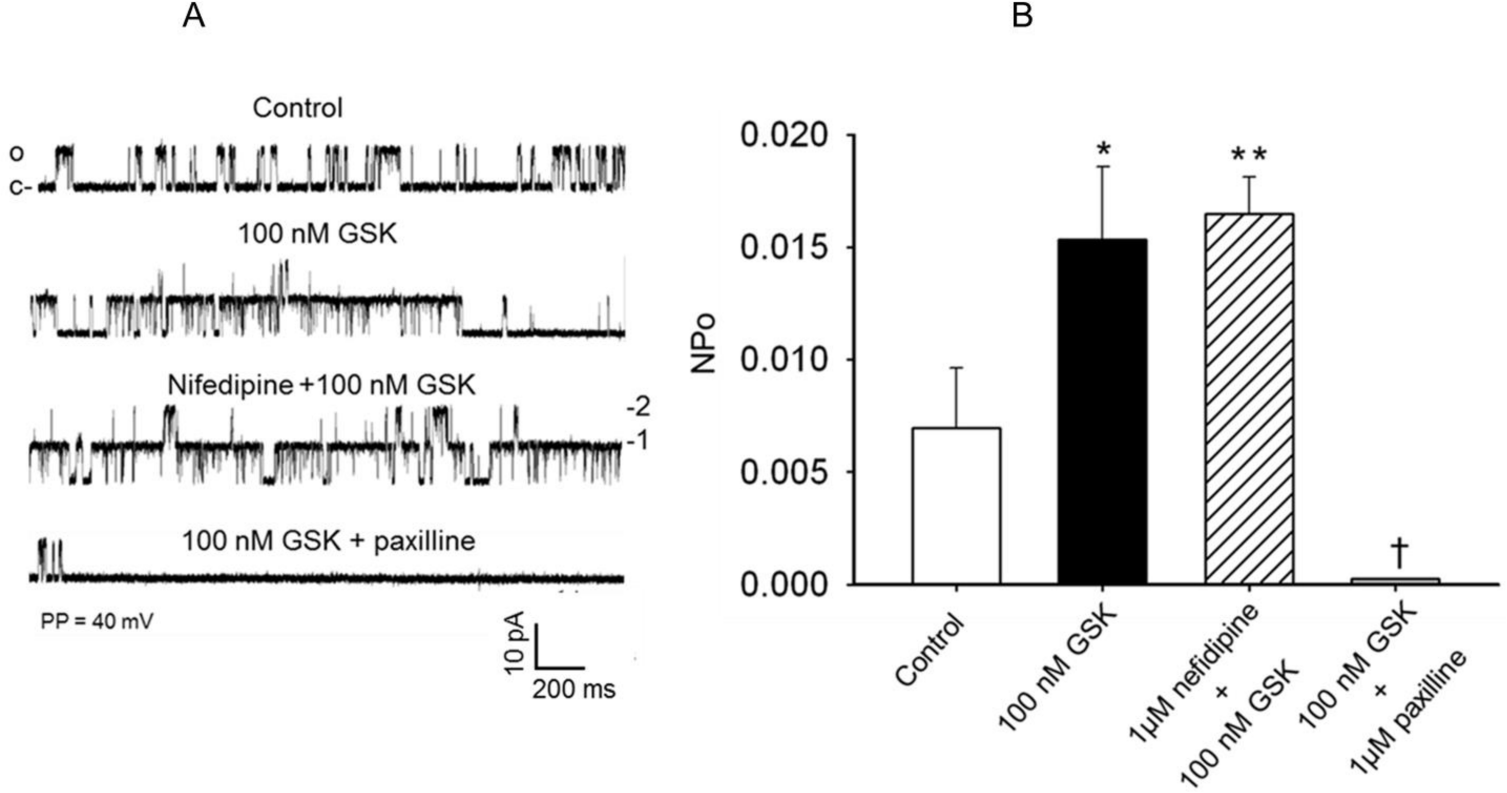
Effect of the L-type Ca^2+^ channel inhibitor nifedipine on the TRPV4 channel agonist GSK1016790A induced activation of K_Ca_ single-channel current in FHH rat cerebral arterial myocytes. (A) Representative K_Ca_ single-channel current traces recorded at a patch holding potential of 40 mV from cell-attached patches under control condition and following treatment with 100 nM GSK1016790A before and after addition of 1 μM nifedipine or the K_Ca_ channel inhibitor 1 μM paxilline to the bath. Treatment with 100 nM GSK1016790A increased the opening frequency of the K_Ca_ single-channel currents that was not influenced by treatment with the L-type Ca^2+^ channel inhibitor 1 μM nifedipine, but attenuated by the K_Ca_ channel inhibitor 1 μM paxilline. (B) Summary of the effects of treatment with 100 nM GSK1016790A on the NPo of the K_Ca_ single-channel currents in the absence and presence of the L-type Ca^2+^ channel inhibitor 1 μM nifedipine or the K_Ca_ channel inhibitor paxilline (1 μM). Treatment with 100 nM GSK1016790A induced a significant increase in NPo of the K_Ca_ single-channel current that was not affected by pretreatment with the L-type Ca^2+^ channel inhibitor 1 μM nifedipine, but attenuated in the presence of the K_Ca_ channel inhibitor 1 μM paxilline. (*,† denote P < 0.05, **denotes P > 0.05, n = 5–6 cells for each group, c and o represent closed and open states of the channel and 1,2,3 indicate stocked channel opening levels).

In additional studies the role of voltage-dependent Ca^2+^ entry in the TRPV4 channel agonist, GSK1016790A, induced activation of K_Ca_ single-channel current was examined using the L-type voltage-gated Ca^2+^ channel inhibitor nifedipine and the T-type voltage-gated Ca^2+^ channel inhibitor nickel (Ni^2+^). In these studies the effects of GSK1016790A on the activities of K_Ca_ single-channel current recorded in cell-attached patches using symmetrical KCl (145 mM) recording solution was assessed in the absence and presence of the L-type Ca^2+^ channel blocker nifedipine (1 μM) or the T-type Ca^2+^ channel inhibitor nickel (Ni^2+^, 50 μM). Treatment of the cell-attached patches with 100 nM GSK1016790A increased the opening frequency and NPo of the K_Ca_ single channel current from the control value of 0.00697 ± 0.00265 to 0.0154 ± 0.00323 (* P < 0.05, n = 5–6 cells) under control condition, and to 0.0165 ± 0.00152 following treatment of the cell-attached patches with 1 μM nifedipine (Fig 8A and 8B, n = 5–6 cells for each group, ** P > 0.05 compared to the effect of GSK1016790A alone).

**Fig 9:**
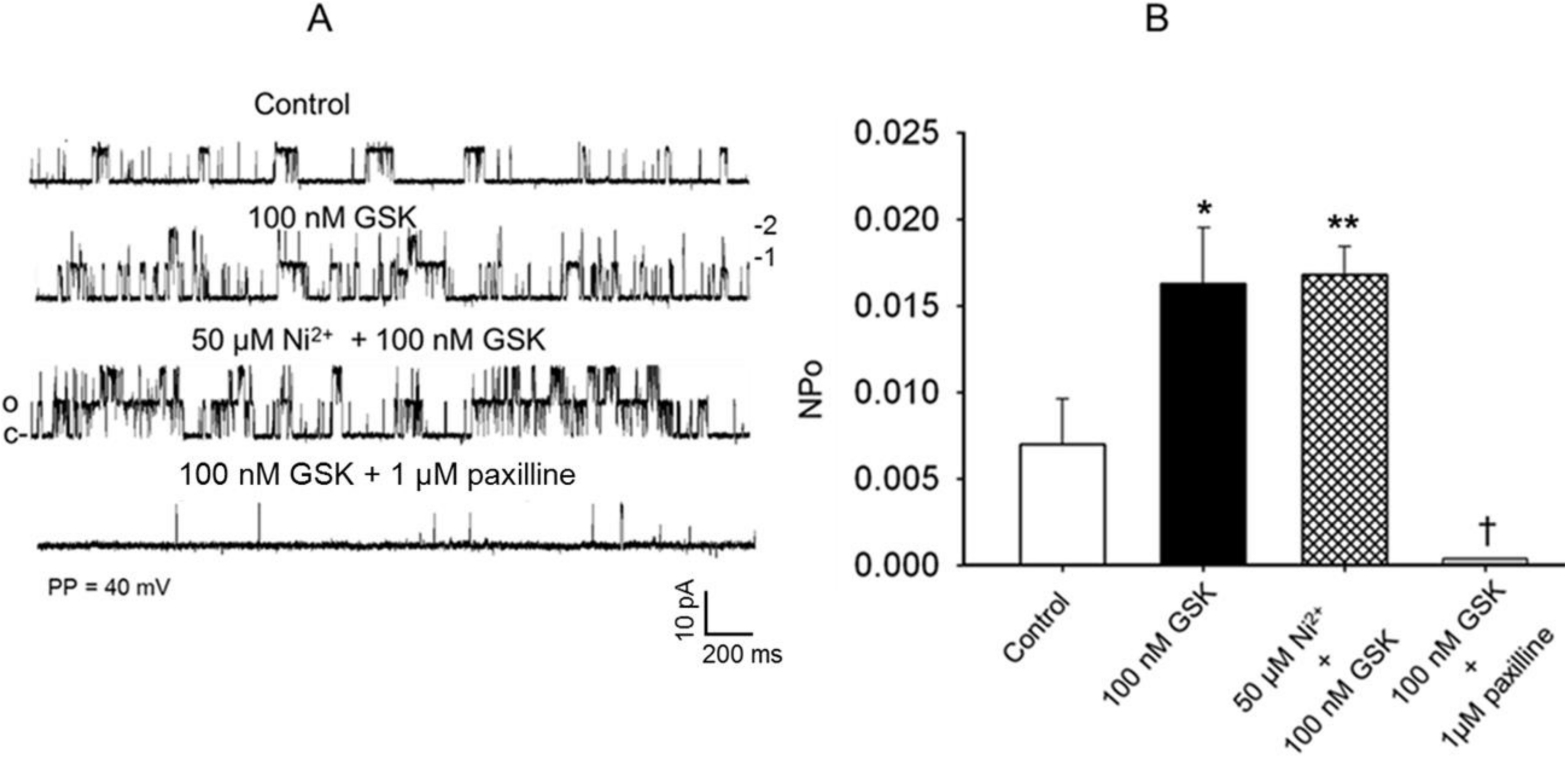
Effect of the T-type Ca^2+^ channel inhibitor nickel on the TRPV4 channel agonist GSK1016790A induced activation of K_Ca_ single-channel current in FHH rat cerebral arterial myocytes. (A) Representative single-channel current traces recorded at a patch holding potential of 40 mV from cell attached patches under control condition and following treatment with 100 nM GSK1016790A before and after addition of the T-type Ca^2+^ channel inhibitor 50 μM nickel (Ni^2+^) or the K_Ca_ channel inhibitor 1 μM paxilline to the bath. Treatment with 100 nM GSK1016790A increased the opening frequency of the K_Ca_ single-channel currents that was not influenced by pretreatment with the T-type Ca^2+^ channel inhibitor 50 μM Ni^2+^ but attenuated by the K_Ca_ channel inhibitor 1 μM paxilline. (B) Summary of the effects of treatment with 100 nM GSK1016790A on the NPo of single-channel K_Ca_ currents in the absence and presence of the T-type Ca^2+^ channel inhibitor 50 μM Ni^2+^ or the K_Ca_ channel inhibitor paxilline (1 μM). Treatment with 100 nM GSK1016790A induced significant increase in NPo of the K_Ca_ single-channel current that was not affected by pretreatment with the T-type Ca^2+^ channel inhibitor 50 μM Ni^2+^, but attenuated in the presence of the K_Ca_ channel inhibitor 1 μM paxilline. (*,† denote P < 0.05, ** denotes P > 0.05, n = 5–6 cells for each group; c and o represent closed and open states of the channel, and 1,2,3 indicate stocked channel opening levels).

As depicted in Fig 9A and 9B treatment of the cell-attached patches with the TRPV4 channel agonist GSK1016790A (100 nM) induced a marked increase in NPo of the K_Ca_ single-channel current from the control value of 0.00721 ± 0.00263 to 0.0164 ± 0.00233 (*P < 0.05) and was not significantly altered in the presence of the T-type Ca^2+^ channel inhibitor 50 μM Ni^2+^ (NPo = 0.0171 ± 0.00162, ** P > 0.05 as compared to the increase in NPo evoked by stimulation with 100 nM GSK1016790A alone).

In these studies the TRPV4 channel agonist GSK1016790A (100 nM) induced increase in opening frequency and NPo of the K_Ca_ single-channel currents was significantly attenuated by application of the selective K_Ca_ channel inhibiter paxilline (1 μM) as shown in Fig 8 or Fig 9 († P < 0.001, n = 5–6 cells for each group). These findings indicate that voltage-dependent Ca^2+^ entry did not appear to involve in the TRPV4 channel agonist induced increase in opening frequency and NPo of the K_Ca_ channel current in the FHH rat cerebral arterial myocytes.

### Effect of inhibition of TRPV4 channel on an increase in intraluminal pressure-dependent reduction in diameter of cerebral arteries

**Fig 10:**
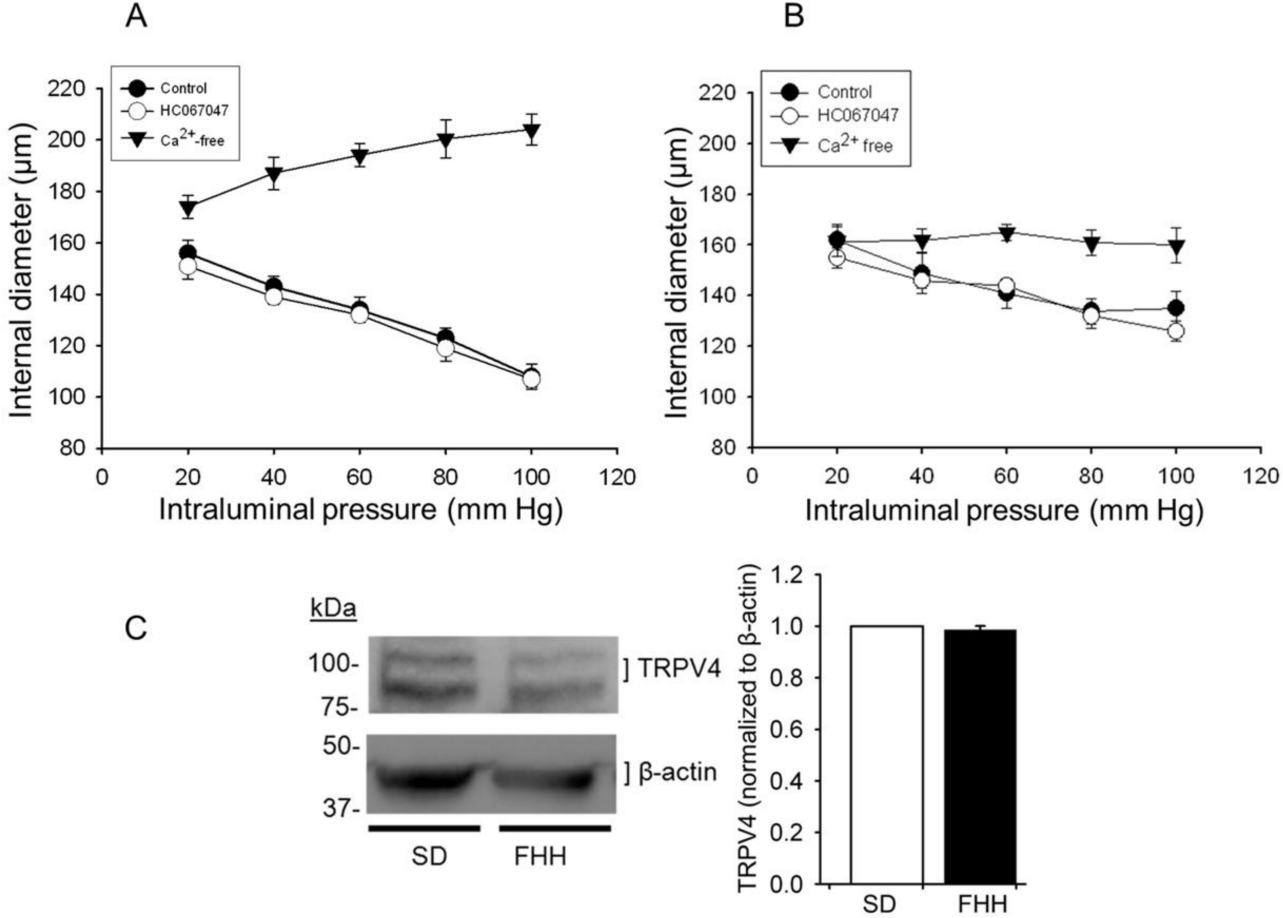
Effects of TRPV4 channel inhibition on intraluminal pressure-dependent reductions in diameter of endothelium-denuded and cannulated cerebral arterial segments of Sprague Dawley (A, SD) and FHH (B, FHH) rats. Pretreatment with the specific TRPV4 channel inhibitor HC067047 (1 μM) had no significant effect on increases in intraluminal pressure induced reductions in diameter of the cerebral arterial segments of SD rat (A) and the FHH rat (B) (n = 5). In Ca^2+^-free PSS bath solution the cannulated cerebral arterial segments of FHH rat exhibited an average passive dilation of 11.2 ± 2.2% whereas the Sprague Dawley rats elicited a mean passive dilation of 31.8 ± 5% (n = 5, < 0.05). Presented in (C) is Western blot showing the level of expression of TRPV4 channel protein with bar graphs depicting summary of the normalized TRPV4 channel protein with the internal control β-actin determined using brain homogenates of SD and FHH rats which are not significantly different (n = 3 independent trials).

The effect of treatment with the specific TRPV4 channel inhibitor HC067047 (1 μM) on step increases in intraluminal pressure-induced reduction in diameter was determined following treatment of endothelium-denuded and cannulated cerebral arterial segments isolated from FHH rat and the control Sprague Dawley rat. Under control condition the cannulated and pressurized cerebral arterial segments of Sprague Dawley rat and the FHH rat maintained or elicited reductions in internal diameter in response increases in intraluminal pressure from 20 to 100 mm Hg in steps of 20 mm Hg (Fig 10A and 10B). Pretreatment with the TRPV4 channel inhibitor HC067047 (1 μM) had no significant effect on the increase intraluminal pressure-induced changes in diameter of the pressurized cerebral arterial segments (Fig 10A and 10B). As depicted in Fig 10A and 10B the pressurized cerebral arterial segments elicited passive increase in diameter in response to elevations in intraluminal pressure from 20 to 100 mm Hg applied in steps of 20 mm Hg confirming development of myogenic tone.

The expression level of TRPV4 channel proteins in brain homogenates of FHH and Sprague Dawley rats was not significantly different as determined by Western blot analysis using TRPV4 specific antibody (Fig 10C). These findings indicate that blockade of TRPV4 channel in cerebral arterial muscle had no significant effect on pressure-dependent reductions in diameter of cerebral arterial segments of FHH rat or the control Sprague Dawley rat. Taken together these findings appear suggest that the TRPV4 channel activity may not be different in the two strains of rats.

## Discussion

The results of the present studies demonstrate presence of TRPV4 channel at mRNA transcript and protein level, and functional properties of TRPV4-like single-channel cationic current activity with unitary slope conductance of 85 ± 2 pS at hyperpolarizing voltages and 96 ± 3 pS at depolarizing voltages in the FHH rat cerebral arterial myocytes (here after: cerebral arterial myocytes). The opening probability of TRPV4-like single-channel cationic current recorded from cerebral arterial myocytes was potently enhanced by the specific TRPV4 channel agonist GSK1016790A and inhibited by two structurally different specific TRPV4 channel antagonists RN 1734 and HC 067074. The TRPV4-like single-channel cationic current recorded in cell-attached patches of cerebral arterial myocytes elicited mechanosensitive property as evidenced by the increased open state probability (NPo) following an increase in membrane stretch induced by application of negative pressure (suction) into the interior of the recording pipette. The observed membrane stretch induced increase in NPo was effectively attenuated by treatment with the TRPV4 channel antagonist HC 067047. Taken collectively, these findings reveal presence of transcript and translated protein for a functional TRPV4 channel in the FHH rat cerebral arterial myocyte that conduct single channel cationic current sensitive to membrane stretch. This TRPV4 channel protein native in the FHH rat cerebral arterial myocyte could be regarded as one of the candidate ion channel targets that could sense and mediate effects of environmental and physical perturbations on the cerebral arterial muscle. The cationic single-channel current recorded in the FHH rat cerebral arterial myocytes in the present study displayed both single-channel conductance and pharmacological properties that resembled those of the reported expressed TRPV4 channel currents [30, 31, 38]. Therefore, it may not be compared or related o the PIEZO 1 channels known to be present in vascular smooth muscle cells and identified to be stretch activated [39].

As also demonstrated in part in the present study (Fig 6), regulation of TRPV4 channel activity is polymodal and is activated by a variety of stimuli including mechanical stretch [1, 10, 12]. However, it is not yet fully settled whether changes in TRPV4 channel current activity could contribute or involve in the initiation or modulation of myogenic vasoconstriction induced by an increase in intravascular pressure. However, there is recent study reporting role of voltage-independent Ca^2+^ entry in mediating low intravascular pressure (< 60 mm Hg)-induced myogenic constriction of endothelium denuded cerebral arterial segments [40]. This intriguing effect was further indicated to occur through increased phosphorylation of myosin light chain 20 (MLC_20_), and was eliminated by treatment with the alkaloid ryanodine that deplete the sarcoplasmic reticulum content of calcium [17, 40]. However, it is not clear from this previous study whether TRPV4 channel activity was involved or not in permitting voltage-independent Ca^2+^ entry that could act to regulate or mediate the reported low intravascular pressure induced cerebral arterial myogenic tone development [40]. In the present study, however, we found that specific inhibition of TRPV4 channel had no significant effect on intraluminal pressure-dependent reduction in diameter of endothelium-denuded and cannulated cerebral arterial segments of FHH rats or the control Sprague Dawley rats in response to increases in intraluminal pressure from 20 mm Hg to 100 mm Hg in steps of 20 mmHg. This apparent lack of inhibition of TRPV4 channel on increases in intravascular pressure-induced cerebral arterial myogenic tone appears to suggest that the TRPV4 channel expressed in cerebral arterial muscle cells is not a mediator of increased intraluminal pressure-dependent myogenic constriction.

One of the goals of the present study was to examine the influence of pharmacological activation of native TRPV4 channel on the opening probability of K_Ca_ single-channel currents recorded in cell-attached patches of FHH rat cerebral arterial myocytes. The results of these studies demonstrated the ability of stimulation with the specific TRPV4 channel agonist GSK1016790A to induce substantial increase in NPo of the K_Ca_ single-channel currents recorded from cell-attached patches bathed in symmetrical potassium (145 mM KCl) solution to prevent possible contribution of fluctuations in cell resting membrane potential. In these studies, stimulation with the TRPV4 agonist induced increased opening of K_Ca_ single-channel currents that was attenuated in the presence of the specific TRPV4 channel inhibitor HC 067047 or the specific K_Ca_ channel blocker paxilline. These findings point to the capacity of increases in activity of native TRPV4 channel to regulate the opening probability (NPo) of K_Ca_ single-channel current thus implicating possible existence of a functional interaction or cooperation between TRPV4 and K_Ca_ channels in the FHH rat cerebral arterial myocytes. The presence of such functional interaction between the TRPV4 channel that could permit voltage-independent Ca^2+^ entry into the arterial myocyte, and the coexisting K_Ca_ channel could be considered to explain the previously reported exaggerated K_Ca_ channel current activity prevailing in the cerebral arterial myocytes of the FHH rat strain as well as the enhanced K_Ca_ channel current activity previously reported in cerebral arterial myocytes of hypertensive rat models [41, 42]. At present involvement of voltage-independent Ca^2+^ entry in inducing activation of the K_Ca_ channel current in the regulation of cerebral arterial tone or reactivity is not completely known. However, such Ca^2+^ entry permitting mechanism could be implicated in the physiological actions of insults such as hypoxia that is known to increase K_Ca_ channel activity and cause hyperpolarization and vasodilation in cerebral arterial myocytes to mediate changes in the dynamics of cerebral blood flow [36, 43].

The observed TRPV4 channel agonist GSK1016790A induced increase in open state probability of the K_Ca_ single-channel currents in the present study could, in part, be related to the reported Ca^2+^ entry through TRPV4 channel evoked localized release of Ca^2+^ from ryanodine-sensitive receptors that resulted in increased openings of spontaneous transient K_Ca_ channel outward currents (STOCs) [17, 44, 45]. Our present finding demonstrating TRPV4 channel activation induced increase in K_Ca_ channel current activity also appears to be in agreement with earlier reports of pharmacological agonist or change in osmolality induced activation of TRPV4 channel that resulted in enhancement of a low conductance K_Ca_ channel current activity recorded in rat hypothalamus slice preparations [5, 6, 46]. On the other hand, the lack of effect of pharmacological inhibitors of voltage-gated Ca^2+^ channel on the TRPV4 channel agonist GSK1016790A induced activation of K_Ca_ channel currents suggests that the TRPV4 channels native in FHH rat cerebral arterial myocytes could function as a voltage-independent Ca^2+^ entry pathway to regulate K_Ca_ channel current activity in these cells. However, the functional contribution of such Ca^2+^ entry pathway to a variety of physiological and pathological processes in the cerebral arterial muscle is unknown and should be explored in future studies.

Interestingly, recent reports indicate that an increase in intracellular Ca^2+^ enabled by voltage-independent Ca^2+^ entry contributes to Ca^2+^ overload in conditions of oxidative stress in which increased expression and function of TRPV4 channel have been reported to result in brain tissue injury or damage and neurotoxicity [47, 48]. In this respect, the observed TRPV4 channel expression and function in the FHH rat cerebral arterial myocytes in the present study could be regarded to serve as a potential molecular target for the investigation of mechanisms of oxidative stress induced injuries and functional abnormalities of the cerebral arterial bed. To date, the only known involvement of TRPV4 channel in cerebral vascular pathologies has been primarily linked to impaired endothelium-mediated cerebrovascular vasodilation [49]. To our knowledge no convincing data in the literature indicative a role for native TRPV4 channels in cerebral pathologies linked to cerebral vascular smooth muscle cell dysfunction. However, there is reported evidence demonstrating an association between of increased TRPV4 channel protein abundance in other vascular beds, such as the bronchial muscle with lung disease, specifically in a condition of pulmonary hypertension [50]. Furthermore, increased TRPV4 expression and function have been found in the rat hippocampal CA1 region of the brain, which in response to exposure to hypoxia and ischemia episodes was found to result in development of astrogliosis and predicted to influence and/or disrupt neurovascular coupling [51]. The TRPV4 channel protein and function detected in the FHH rat cerebral arterial myocytes in the present study could be considered to operate as a potential pathway permitting voltage-independent Ca^2+^entry. In this regard, unraveling changes in TRPV4 channel expression and activity in cerebral arterial muscle pathologies of humans and animal disease models may lead to the development of TRPV4 channel targeted therapeutics to manage associated functional disorders of the cerebral circulation.

## Viewpoint

The FHH rat has been previously identified (found) to possess a disrupted pressure-dependent cerebral arterial myogenic response and elevated systemic arterial pressure. The disrupted cerebral arterial myogenic response has been previously suggested to enhance transmission of systemic pressure to the cerebral microcirculation and to exacerbate brain tissue injury following ischemia reperfusion. The signaling mechanisms of cell reaction to oxidative stress are not completely understood and are increasingly important focus of investigation. The TRPV4 channel, known to permit voltage-independent Ca^2+^ entry, has been implicated in the pathogenesis of tissue injury in response to oxidative stress. The presence of a functional TRPV4 channel protein coupled to the K_Ca_ channel activity in the FHH rat cerebral arterial myocytes could be targeted as a potential molecular therapeutic entity for management of cerebral microvascular brain injury resulting from Ca^2+^overload associated with oxidative stress.
